# Deep cutaneous candidiasis of the lip in a patient with acute myelogenous leukemia

**DOI:** 10.1016/j.jdcr.2022.06.039

**Published:** 2022-07-08

**Authors:** Jose L. Cortez, Sally Y. Tan, Rebecca Abelman, Peter Chin-Hong, Timothy H. McCalmont, Lindy Fox, Anna Haemel

**Affiliations:** a Department of Dermatology, University of California, San Francisco, San Francisco, California; b Department of Dermatology, University of New Mexico, Albuquerque, New Mexico; c Department of Pathology, University of California, San Francisco, San Francisco, California; d Golden State Dermatology Associates, Walnut Creek, California

**Keywords:** acute myelogenous leukemia, *Candida dubliniensis*, deep cutaneous candidiasis, fluconazole, hard palate, necrotic plaque, vermillion lip

## Introduction

Mucocutaneous lesions may be the presenting sign in superficial and deep cutaneous fungal disease, presenting with various morphologies ranging from papulopustular to necrotic papules or plaques. Invasive candidiasis typically presents as disseminated acneiform-appearing papules in a patient with fevers and myalgias.^1^^,^^2^ Here, we discuss a case of deep cutaneous candidiasis presenting as a single indurated necrotic plaque of the upper vermillion lip, with the clinical presentation initially more suggestive of mucormycosis.^3^

## Case report

**Fig 1:**
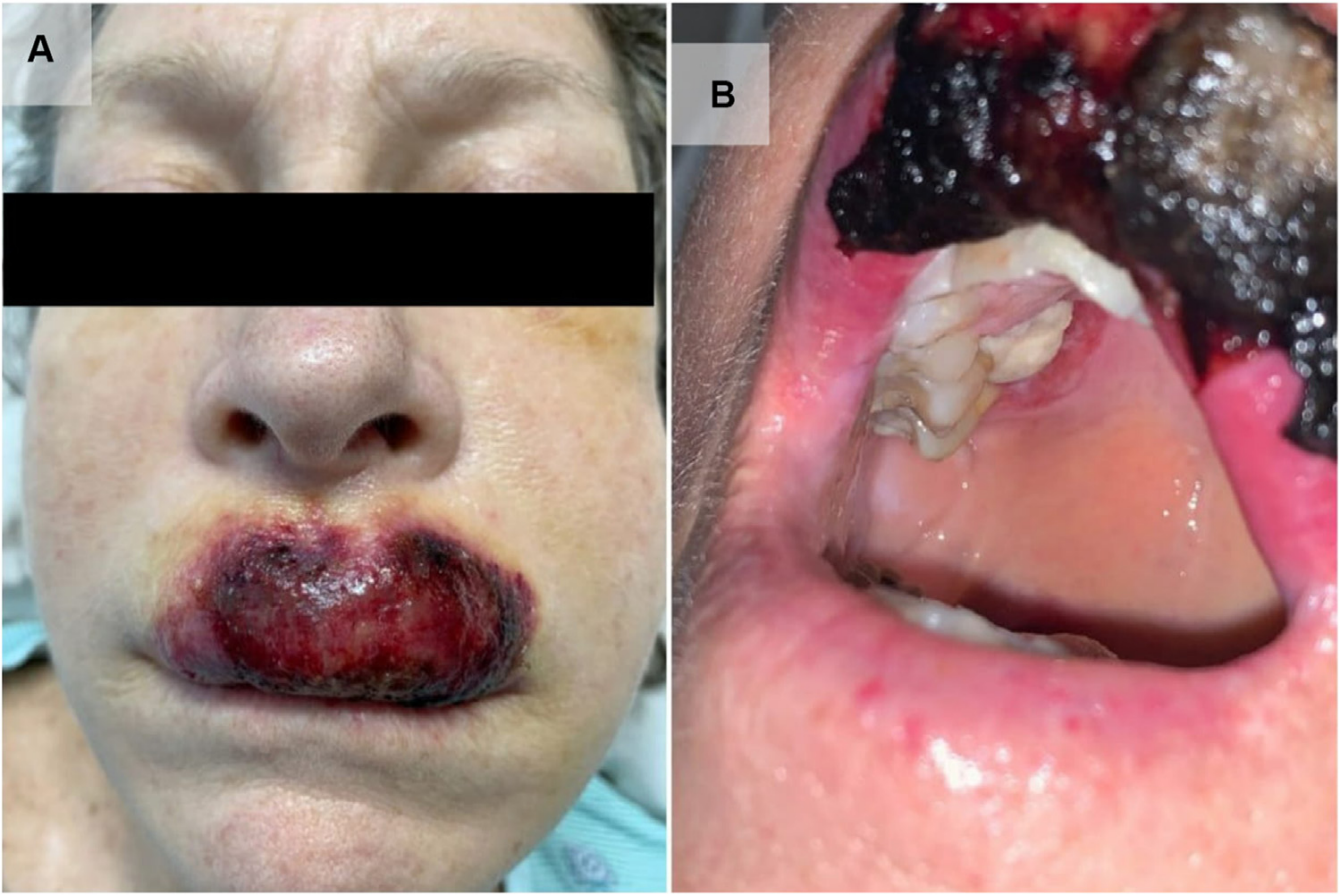
Clinical presentation of deep cutaneous candidiasis of the lip. **A,** Indurated ulcerating purpuric plaque involving the upper vermillion lip. **B,** Spontaneous ulceration and bleeding of lip lesion; presence of angulated and ulcerated plaque on hard palate with violaceous rim.

A 56-year-old female with acute myeloid leukemia was admitted for induction chemotherapy with cytarabine and daunorubicin. Dermatology was consulted for an indurated purpuric ulcerating plaque of the upper vermillion lip that had been tender and enlarging for 2 weeks (Fig 1, *A*). On examination, the patient was also noted to have a painful angulated ulcerated plaque with a violaceous rim on her hard palate (Fig 1, *B*). She had no history of orolabial herpes simplex virus infection. The differential diagnosis included infiltrative leukemic involvement of skin/mucosa, lymphoma, melanoma, neutrophilic dermatosis, and invasive fungal infection.

**Fig 2:**
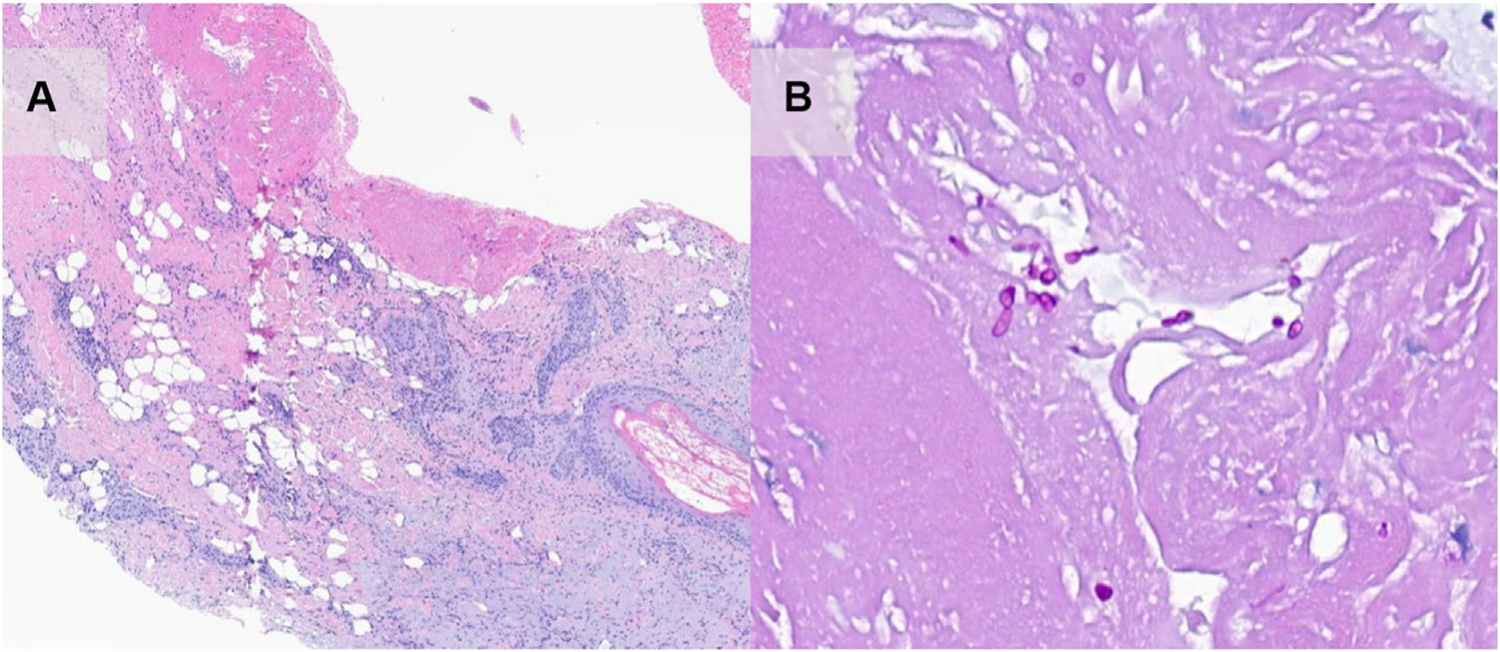
Dermatopathology findings of deep cutaneous candidiasis of the lip. **A,** Sections stained with hematoxylin and eosin demonstrate a necrotizing panniculitis (magnification ×20). **B,** In the deep necrotic area, periodic acid Schiff plus diastase staining reveals budding yeast forms with pseudohyphae suggestive of *Candida* morphology. No angioinvasion is apparent.

A skin biopsy of the vermillion lip was performed, with amphotericin B initiated thereafter given clinical concern for mucormycosis. A potassium hydroxide preparation performed on skin tissue showed numerous pseudohyphae and yeast forms. Dermatopathologic examination revealed deep periodic acid Schiff plus diastase + budding yeast forms suggestive of *Candida* species without angioinvasion (Fig 2). Tissue culture grew numerous *Candida dubliniensis*. Beta-d-glucan and galactomannan tests were both negative. Admission blood cultures remained negative. The patient was transitioned to voriconazole once susceptibilities returned with complete resolution over 3 months.

## Discussion

Primary deep cutaneous candidiasis describes a rare phenomenon in which candida is found in deeper cutaneous structures but has not yet disseminated.^4^ Invasive candidiasis comprises both candidemia and deep-seated candidiasis.^1^ Though candidemia is generally more common, deep-seated disease may arise from either direct inoculation of a sterile site or hematogenous spread.^1^^,^^5^ Invasive candidiasis should be considered in patients with neutropenia and have unexplained fever unresponsive to antibiotics.^6^^,^^7^ Ocular involvement is common in disseminated disease and should be evaluated when there is a suspicion and/or when ocular symptoms are present.^7^ Our patient presented with a deep cutaneous infection as shown by her biopsy, and her negative blood cultures and lack of additional symptoms and organ involvement suggested her disease was not yet invasive.

Diagnosing deep cutaneous candidiasis involves biopsy with tissue culture to isolate the fungal organism.^1^^,^^8^ Historically, *albicans* species accounted for the majority of isolates. However, nonalbicans *Candida* species are becoming increasingly prevalent and account for about half of all isolates detected in recent surveys.^1^^,^^5^ Our patient’s tissue culture grew *C dubliniensis*, a species first described in 1995 that is seen in immunocompromised hosts, is associated with fluconazole resistance, and is similar to *Candida albicans*.^9^ *Candida* species are generally treated with azoles though this class is not recommended as initial therapy in patients with neutropenia given possibility of azole-resistant candida species or other nonresponsive organisms.^5^ Instead treatment should be initiated promptly with broad-spectrum agents such as amphotericin B and narrowed if and when appropriate, such as with our patient who completed her course on voriconazole.^5^

Herein, we described a case of primary deep cutaneous candidiasis in a patient with neutropenia presenting as a single indurated, ulcerated plaque mimicking mucormycosis rather than the more typical diffuse erythematous papular eruption often seen in disseminated disease^1^^,^^2^ or white patches seen in superficial oral disease. To the best of our knowledge, this individual represents the first case of deep cutaneous candidiasis with *C dubliniensis* in an oncology patient. Although there is no specific amount of time before dissemination can be expected, it is important not to delay treatment as 1-2 day delays in initiation of therapy portend increased mortality.^6^ While initial antifungal treatment for the clinical presentation and morphology presented herein should still include coverage for mucormycosis molds, patients confirmed to have candidiasis alone after careful clinicopathologic correlation can be narrowed to azoles or echinocandins upon speciation. Ultimately, our patient’s cultures were susceptible to azoles, and she did well on voriconazole alone.

## Conflicts of interest

Haemel serves as a consultant to CSL Behring and Guidepoint LLC. Cortez, Tan, Abelman, Chin-Hong, McCalmont, and Fox have no conflicts of interest to disclose.
